# Aconiti Lateralis Radix Praeparata ameliorates heart failure via PI3K/AKT/Bnip3 pathway

**DOI:** 10.3389/fphar.2025.1526653

**Published:** 2025-03-26

**Authors:** Wenxiu Liu, Xingju Zou, Yang Zheng, Yuan Zhang, Guijuan Cui, Siyu Liu, Chen Sun, Cheng Peng

**Affiliations:** State Key Laboratory of Southwestern Chinese Medicine Resources, Chengdu University of Traditional Chinese Medicine, Chengdu, China

**Keywords:** Aconiti Lateralis Radix Praeparata, transverse aortic constriction, chronic heart failure, network analysis, metabolomics

## Abstract

**Background:**

Chronic heart failure (CHF) is one of the leading causes of high mortality worldwide. It is characterized by pathological hypertrophy and poses a major threat to human health. Aconiti Lateralis Radix Praeparata is widely used in ancient China to treat CHF. However, the pathology is obscured, necessitating further exploration.

**Methods:**

Prospective targets were predicted by network analysis. A transverse aortic constriction (TAC) mice model was subsequently constructed to determine the effects of aqueous extract of Aconiti Lateralis Radix Praeparata (AEA) on CHF. The echocardiography was performed to investigate cardiac function. Histopathological analysis of cardiac tissue was conducted to assess myocardial fibrosis. Nontargeted metabolomics was performed to analyze serum metabolites. The phosphorylation level of PI3K and AKT, and downstream targets such as Bnip3, p62, Atg5, and LC3II were measured by Western blotting. *In vitro*, norepinephrine (NE) was used to stimulate cardiac hypertrophy. Parameters such as reactive oxygen species levels, mitochondrial membrane potential, ATP concentration, and CK/MB content were detected in H9c2 cells.

**Results:**

AEA significantly alleviated CHF. Network analysis indicated the participation of AKT in CHF, and was modulated by Aconiti Lateralis Radix Praeparata. *In vivo*, AEA administration effectively ameliorated cardiac performance, evidenced by the elevation of ejection fraction. Histopathological analysis displayed a diminishment of collagen fiber. Metabolomics analysis showed that several metabolites such as tetrahydroxycorticosterone, decylubiquinone and taurocholic acid were increased in the TAC mice serum. Additionally, the phosphorylation levels of PI3K and AKT, and expression levels of Drp1, Opa1, Bnip3, p62, Atg5 and LC3II were altered in TAC group. *In vitro*, NE stimulation increased the cell surface area and deteriorated mitochondrial functions in H9c2 cells. However, AEA administration partially reversed such results, and the mechanism was associated with mitophagy.

**Conclusion:**

This study revealed that AEA improved cardiac function via the PI3K/AKT/Bnip3 pathway.

## 1 Introduction

Chronic heart failure (CHF) is a progressive disease that affects cardiac function and morphology with multiple etiologies (e.g., hypertension, myocardial infarction, diabetes, obesity, pregnancy, and doxorubicin overuse) (Zhang H. et al., 2024). The incidence of CHF has exponentially increased, with 64 million people worldwide, leading to a major threat to human health (Li X. et al., 2024). Although great advances have been achieved in surgery, transplantation, and drug administration, the 5-year survival rate remains dismal, with estimates of no more than 50% (Dong et al., 2024). Drug administration is the most common treatment, and aldosterone, enkephalinase inhibitors, angiotensin receptor inhibitors, angiotensin receptor blockers, angiotensin-converting enzyme inhibitors, and β-blockers are certified as “new quadruple drugs” (Zhou et al., 2024). However, the prospects are still not optimistic, certain effects such as renal damage and hyperkalemia emerge after long-term administration (Ali et al., 2023). Thus, more endeavors are needed in new drug exploration. Traditional Chinese medicine (TCM) has been employed as adjuvant therapy in the medical field, and its secondary metabolites are the primary source of new pharmaceutical drugs (Yu et al., 2024). Thus, it is necessary to explore the compounds and associated effects (Chen et al., 2023).

Mitochondrial damage is commonly seen in the development of CHF, primarily caused by detrimental stimulation and consequently disturbed energy metabolism (Hinton et al., 2024). It may provoke programmed cell death, calcium excess, phagocytosis abnormalities, oxidative stress, energy metabolism complications at the intracellular level, and impaired mitochondrial biosynthesis (Lawson et al., 2024). Bcl-2/adenovirus E1B 19 kDa protein-interacting protein 3 (Bnip3), a member belonging to the BH3 family, is reported to enhance mitophagy upon adverse stimulation. (Liu et al., 2024). Several factors such as mTOR, FOXO, and HIF-α were reported to regulate Bnip3 expression, and these proteins were modulated by the PI3K/AKT axis. Previous studies suggested that the activation of the PI3K/AKT reduced Bnip3 expression and improved mitochondrial biological functions in cardiac injuries (Li Y. et al., 2024). Moreover, Bnip3 was tightly associated with mitochondria and exerted a prominent role in membrane permeability, cytochrome C release, and endogenous apoptosis (Liu et al., 2016). Mitophagy eliminates injured mitochondria via the combination of autophagosomes and lysosomes and cooperates with fusion, fission, and biogenesis to maintain mitochondrial dynamics (Abbasi Moajani et al., 2025). The process can be briefly summarized as follows: adverse stimulation induced mitochondrial damage thereby promoting asymmetrical fission. Then, Bnip3 was activated, and autophagosomes were recruited via the LC3-interacting region (LIR). Finally, through the interaction of lysosomes, the content was degraded via different hydrolases (Zhu et al., 2020).

Aconiti Lateralis Radix Praeparata, the lateral root of *Aconitum carichaelii* Debx., is traditional Chinese medicine, with the effects of restoring yang, relieving stress, tonifying fire, and dispersing cold (He et al., 2023). It was extensively used in ancient China to treat acute and chronic heart failure, and the mechanisms were associated with cell apoptosis, hypertrophic factors, calcium transport, mitochondrial respiration, and inflammatory response (Xiang et al., 2024). It contains a variety of alkaloids with potent effects on cardiac performance, such as salsoline, songorine, aconitine, hypaconitine, and mesaconitine, among which water-soluble alkaloids are principal in cardiac functions. Salsolinol was a tetrahydroisoquinoline alkaloid extracted from Aconiti Lateralis Radix Praeparata with cardiotonic effects, as well as altered the metabolic rate of neurohumoral metabolism by affecting catecholamines synthesis in organisms (Wang et al., 2025a). Moreover, salsolinol was a weak β-adrenergic receptor agonist that affected systolic blood pressure, myocardial contraction frequency, and contractility (Wen et al., 2019). Songorine was a napelline-type alkaloid, which promoted the binding of PGC-1α, and alleviated septic cardiomyopathy via intraperitoneal injection at 50 mg/kg (Li et al., 2021). Specifically, songorine was reported to activate SIRT1, inhibit its transfer, and promote mitochondrial biogenesis (Li, et al., 2021). Mesaconitine was a diester alkaloid that alleviated doxorubicin-induced mitochondrial damage via Pink1-mediated mitophagy (Zhou et al., 2023). Therefore, Aconiti Lateralis Radix Praeparata alleviates CHF symptoms by restoring mitochondrial functions through different compounds.

Network analysis is an analytical method involving the similarity of molecular structure and functions that use existing database resources to predict drug-disease associations (Shang et al., 2025). Network pharmacology is a comprehensive and systematic method, which is consistent with the TCM holistic view (Dai et al., 2024). However, the databases are not yet mature and have some drawbacks. Moreover, network pharmacology is mostly based on previous research foundations, and its innovation and reliability are questionable (Fu et al., 2023). Thus, it’s necessary to validate the predictions via experiments.

Our previous studies revealed that AEA could affect substance metabolism in CHF by influencing GSK3β expression, a downstream target of AKT (Xing et al., 2024). In this study, we intend to clarify the effectiveness of the aqueous extract of Aconiti Lateralis Radix Praeparata (AEA) on Bnip3-mediated mitophagy.

## 2 Materials and methods

### 2.1 AEA preparation

Aconiti Lateralis Radix Praeparata was purchased from Sichuan Jiangyou Zhongba Aconite Technology Development Co., Ltd. The detailed preparation method and compound analysis can be found in our previous study (Xing et al., 2024). In short, after soaking for 30 min, the medicinal herb was boiled for 5 h. Filtered and then boiled for another 3 h. Concentrating the final liquid and detecting the compounds via a mass spectrum.

### 2.2 Materials

Norepinephrine (N7960), and Hematoxylin & Eosin (H&E, G1120) kits were purchased from Beijing Solarbio Co., Ltd. (Beijing, China). Anti-phospho-AKT (Ser473) antibody, DCFH-DA (S0034S), and JC-1 (C2003S) were purchased from Beyotime (Beijing, China). Hoechst 33,342 (70100500) was purchased from Biosharp (Hefei, China). Masson’s trichrome kit (G1006) and RIPA buffer (G2002) were purchased from Servicebio (Wuhan, China). N-terminal pro-brain natriuretic peptide kit (NT-pro-BNP, E-EL-M0834c), creatine kinase MB kit (CK-MB, E-EL-M0355c), and anti-GAPDH (E-AB-48016) were purchased from Elabscience (Wuhan, China). Anti-LC3B (BM4827), anti-Bnip3 (BM5174), anti-p62 (PB0458), anti-Atg5 (PB9076), anti-Drp1 (A00556), and anti-Opa1 (A00508) antibodies were purchased from Boster (Wuhan, China). Anti-AKT (A17909), anti-phospho-PI3K (AP0427) antibodies were purchased from Abclonal (Wuhan, China). Anti-PI3K antibody (25868-1-AP) was obtained from Proteintech (Wuhan, China). Anti-MMP2 (#AF5330), and anti-MMP9 (#AF5228) were brought from Affinity (Jiangsu, China). Anti-Trx2R (YT4752) antibody was obtained from Immunoway (Texas, United States).

### 2.3 Network analysis

#### 2.3.1 Target prediction

Ingredients of Aconiti Lateralis Radix Praeparata were downloaded from the TCMSP database https://www.tcmsp-e.com/tcmsp.php and filtered ingredients. SMILES numbers for ingredients of Aconiti Lateralis Radix Praeparata were downloaded from the PubChem database (https://pubchem.ncbi.nlm.nih.gov) and then imported into the SwisstargetPrediction database (https://swisstargetprediction.ch/). These targets were collected and filtered for subsequent analysis.

#### 2.3.2 Disease target prediction

Using “chronic heart failure” to search for target genes in the GeneCards (https://www.genecards.org/), DisGeNET (https://www.disgenet.org/), and OMIM (https://www.disgenet.org/). The median number was used to control the target number.

#### 2.3.3 PPI network

The data were processed via STRING 11.5 data platform (https://string-db.Org/). “*Homo sapiens*” was selected, and interactions were obtained. Cytoscape 3.9.1 software constructed the PPI network. The ‘Network Analyzer’ tool was used to determine the degree value, closeness centrality, and betweenness centrality.

#### 2.3.4 KEGG analysis and GO analyses

Targets were imported into DAVID 2021 database (https://david.ncifcrf.gov/). Using “*Homo sapiens*” as a species, selecting the top ten objects with *P* < 0.05. DAVID database was used to identify target genes on the KEGG pathway and GO annotation (Sherman et al., 2022).

### 2.4 *In vivo* experiments

#### 2.4.1 TAC surgery

Four-week-old C57BL/6 male mice were purchased from Chengdu Dossy Experimental Animal Company with a license (NO. SCXK 2020-030). 40 C57BL/6 male mice with similar health conditions were divided into a Sham (10 mice) group and a model group (30 mice). The experimental procedures were approved by the Ethics Committee of Chengdu University of Traditional Chinese Medicine (No. 2023020). Anesthesia was induced with 50 mg/kg sodium pentobarbital and the hair was removed for skin preparation. A chest cavity was made from the second intercostal space. The transverse aortic arch was exposed for ligation. A customized hook was used to expose the aorta, which was subsequently ligated with 6-0 nylon thread. The narrowing degree is determined with a 27-size needle. Sham group underwent the same procedures except for narrowing. The wound was sutured, and mice were transferred to a heating pad at 37°C.

#### 2.4.2 Grouping

One week after surgery, C57BL/6 mice were randomly divided into the Sham, TAC, high-dose AEA group (5 g/kg) and low-dose AEA groups (2 g/kg). Continuous gavage was performed for 21 days. According to the requirements of *Chinese Pharmacopoeia*, the maximum daily dosage for adults is 15 g. According to the *Experimental Course of Pharmacology of Traditional Chinese*, based on a 70 kg adult body weight, the daily dose for mice is approximately 2.5 g/kg. Thus, in our experiment, we set up two dose groups based on raw pieces, with a high dose at 5 g/kg and a low dose at 2 g/kg. Mice in Sham and TAC groups were administered with the same volume of 0.9% physiological saline.

#### 2.4.3 Echocardiography

Three weeks after surgery, C57BL/6 mice were anesthetized with isoflurane and depilated. An MX 550 probe was used to assess the cardiac function. Long-axis images were recorded in M-mode. A Vevo 3100 high-resolution ultrasonic system (FUJIFILM VisualSonics) was used to analyze cardiac parameters and calculate the final data at least 3 cycles.

#### 2.4.4 H&E staining

Mice were sacrificed, and the cardiac tissues were fixed with 4% paraformaldehyde. The heart tissue was subsequently dehydrated with gradient alcohol solutions (70%, 80%, 90%, and 100%). Then, the heart mass was embedded in paraffin and cut into 5 µm slices. Hematoxylin staining was performed for 1 min, differentiated by 0.5% hydrochloric acid, and observed under a microscope. The differentiation fluid was removed with running water. Performing eosin staining for 1 min and removing excess dye. Subsequently, the heart slices were sealed with neutral gum, and dried for 24 h. Finally, the heart slices were scanned for analysis.

#### 2.4.5 Masson’s trichrome stain

Heart tissue was gradient rehydrated and stained by hematoxylin, differentiated with 0.5% hydrochloric acid ethanol, and washed with running water. Conducted ponceau red staining for 5 min, and performed phosphomolybdic acid treatment for 5 min. Aniline blue was applied for 5 min, and 1% glacial acetic acid treatment was performed for 1 min. Gradient dehydration and sealed. The final slices were scanned for analysis.

#### 2.4.6 ELISA determination of serum factors

Blood was collected from the mice and left for 2 h. Then, centrifuged at 3,500 r/min for 15 min, and collected the upper serum. According to the instructions of reagent kits, the concentrations of NT-pro-BNP and CK-MB were measured separately.

#### 2.4.7 Metabolomics analysis

Adding acetonitrile: methanol (1:1) to mouse serum at a ratio of 1:4. The mixture was inverted and mixed 20 times, vortexed for 30 s, sonicated for 20 min, and stored in the refrigerator for 30 min. Then, the samples were centrifuged at 12,000 rpm for 15 min and the supernatant was extracted. Metabolite ion fragments were collected by the AB SCIEX ZenoTOF 7600 system. Mobile phase A (0.1% formic acid), and phase B (0.1% formic acid acetonitrile). The flow rate was 0.3 mL/min. Gradient elution was set as follows: 0–5 min, 20:80, v/v; 5–8 min, 20:80 to 30:70, v/v; 8–10 min, 30:70 to 40:60, v/v; 10–13 min, 40:60 to 60:40, v/v; 13–15 min, 60:40 to 80:20 v/v. Fragments were collected in different ion modes. The mass spectrometry data were processed with MExplore software.

#### 2.4.8 Western blotting

RIPA buffer was used to extract the total protein and measure concentration. SDS polyacrylamide gel electrophoresis was used to separate proteins. A PVDF membrane was activated with methanol for 1 min, transferring target proteins for 1 h, and adding corresponding PI3K, p-PI3K, AKT, p-AKT, Bnip3, Atg5, LC3, p62, Opa1, Drp1 primary antibodies overnight. The second antibody was incubated at room temperature. Protein bands were visualized via an ECL chemiluminescent reagent.

#### 2.4.9 Immunohistochemistry

After the paraffin sections were gradually rehydrated, they were repaired with antigen retrieval solution, then inactivated with 3% hydrogen peroxide solution for 5 min, incubated overnight with primary antibody, washed off with running water, secondary antibody was added, DAB differentiated, and hematoxylin stained the nucleus.

#### 2.4.10 PCR analysis

Total RNA in heart tissue was extracted via Trizol and reverse transcribed into cDNA via Evo M-MLV RT Mix Kit. An SYBR Green Pro Taq HS mix kit was employed to detect Nppa, Nppb, Tgfb1, and Bnip3 levels, specific primers were listed in Table 1.

**TABLE 1:** Primer sequence.

Gene	Forward sequence	Reverse sequence
Nppa	AGC​CGT​TCG​AGA​ACT​TGT​CTT	CAG​GTT​ATT​GCC​ACT​TAG​GTT​CA
Nppb	GAG​TCC​TTC​GGT​CTC​AAG​GC	TAC​AGC​CCA​AAC​GAC​TGA​CG
Tgfb1	AGG​GCT​ACC​ATG​CCA​ACT​TC	CCA​CGT​AGT​AGA​CGA​TGG​GC
Bnip3	GGA​TAT​GGG​ATT​GGT​CAA​GTC​GA	GTT​GTC​AGA​CGC​CTT​CCA​ATG​TA
Gapdh	GAA​GGT​CGG​TGT​GAA​CGG​AT	CCC​ATT​TGA​TGT​TAG​CGG​GAT

### 2.5 *In vivo* experiments

#### 2.5.1 MTT assay

H9c2 cells were seeded at 8000 per well and incubated overnight. Discarding the 10% DMEM culture medium from the wells and dividing the cells into 6 groups. Each group was given different concentrations of drug-containing serum (5%, 4%, 3%, 2%, 1%), with 5 wells per group. Cultivating the cells for 24 h. MTT solution was added, cultured for another 4 h, and all liquid was discarded. Then, 150 µL DMSO solution was added, and the OD value was measured.

#### 2.5.2 Cell surface area evaluation

H9c2 cells were stimulated with NE and incubated with AEAH (1%), and AEAL (0.5%) for 24 h. Rinsed with PBS thrice, fixed by paraformaldehyde, permeabilized for 5 min, blocked with BSA for 2 h, incubated with CY3 labeled anti-α-actin antibody for 2 h, and stained with DAPI dye for 40 min. Finally, the samples were photographed with a fluorescence microscope.

#### 2.5.3 ROS detection

H9c2 cells were seeded and then treated with corresponding agents for 24 h. Then, cells were collected with 0.25% trypsin and rinsed with PBS thrice. DCFH-DA was applied for 20 min to sustain the cells, and the excess agent was removed with PBS. Flow cytometry was utilized to detect the fluorescence intensity.

#### 2.5.4 Mitochondrial membrane potential detection

H9c2 cells were seeded and treated for 24 h. The surplus culture medium was discarded and washed thrice with PBS. Adding 1 mL JC-1 dye to each well and incubating for 20 min. Images were captured via a fluorescence microscope.

#### 2.5.5 ATP detection

An enhanced assay kit was used to quantify ATP levels in H9c2 cells. Briefly, H9c2 cells were collected and lysed in a lysis solution. 200 μL of the liquid was scraped and pipetted into EP tubes. A 20 µL aliquot of supernatant was used to prepare the ATP solution. A luminometer device was used to detect light units.

#### 2.5.6 Transmission electron microscope

DMEM was discarded to obviate the effects of 2.5% glutaraldehyde. Fixed by 2.5% glutaraldehyde for 4 h. 1% osmium tetroxide was used for rear fixation and then dehydrated. 60 nm slices were prepared for the transmission electron microscope.

#### 2.5.7 ELISA determination of serum metabolites

H9c2 cells were seeded and treated with corresponding agents for 24 h. Then, the samples were collected and the mitochondria were isolated with a separation kit for the following detection. The concentration of CK/MB was detected according to the instructions.

#### 2.5.8 Immunofluorescence

H9c2 cells were harvested and fixed with 3.7% paraformaldehyde and rinsed with PBS twice. Drilled by 0.1% triton X-100 (Art. No.ST797, Beyotime, CHA), and incubated with Bnip3, Drp1 or p62 antibody for 12 h. FITC-labeled goat anti-rabbit IgG (H + L) or Cy3-labeled goat anti-rabbit IgG (H + L) was conjugated with the first antibody for 2 h, along with DAPI. The remaining fluorescence dye was removed by PBS. Images were captured via a confocal microscope.

#### 2.5.9 Western blotting

H9c2 cells were lysed in a lysis buffer supplemented with 1% protease and phosphatase inhibitors. The cells were incubated on ice for 30 min and then collected in EP tubes. Then, the supernatant was centrifuged at 12,000 r/min for 15 min 4% loading buffer was added and boiled at 100°C for 5 min. The subsequent steps were similar to those described in Section 2.4.8.

### 2.6 Data analysis

All the results of these experiments were analyzed via SPSS 26.0, and the bar charts were got from GraphPad Prism 9.0. Results were presented as mean ± SD. One-way ANOVA analysis was performed if the data was normally distributed. Otherwise, non-parametric tests will be carried out. *P < 0.05* was considered to have a significant difference.

## 3 Results

### 3.1 Compounds analysis

**FIGURE 1:**
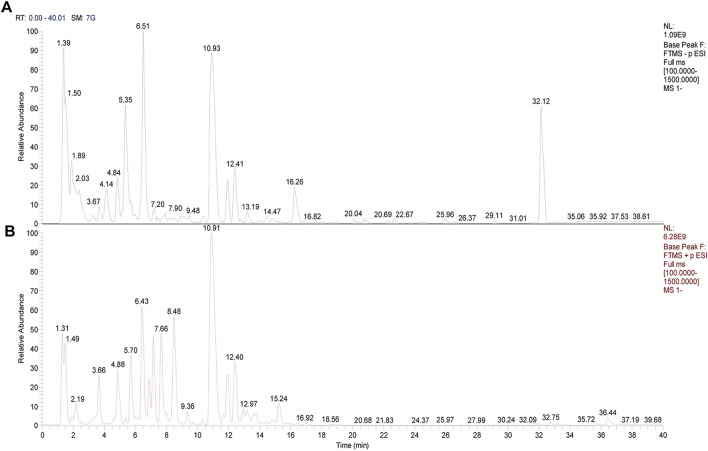
Mass spectrum image of compounds in aqueous extract of Aconiti Lateralis Radix Praeparata (AEA). **(A)** Negative mode. **(B)** Positive mode.

UPLC-Q-Orbitrap-HRMS was used to detect the components of AAE. Thousands of chemical compounds were identified in both positive and negative ionizations (Figures 1A, B). Seven alkaloids were identified and matched in AAE powder, as detailed in Table 2.

**TABLE 2:** Compounds of AEA.

No.	Components	Retention time (min)	Molecular formula	Detected (m/z)	Theoretical mass	Error (ppm)
1	Benzoylmesaconitine	10.919	C_31_H_43_NO_10_	589.28789	590.29516	−1.37
2	Talatisamine	7.654	C_24_H_39_NO_5_	422.28985	421.28258	−0.58
3	Neoline	7.147	C_24_H_39_NO_6_	438.28503	437.27776	0.05
4	Benzoylhypaconitine	12.402	C_31_H_43_NO_9_	574.30061	573.29334	−0.78
5	14-Benzoylaconitine	11.932	C_32_H_45_NO_10_	603.30384	604.31111	−0.84
6	Aconitine	5.657	C_25_H_41_NO_9_	499.27781	500.28508	−0.65
7	Mesaconitine	14.203	C_33_H_45_NO_11_	631.29967	632.30694	0.64

### 3.2 Network analysis

**FIGURE 2:**
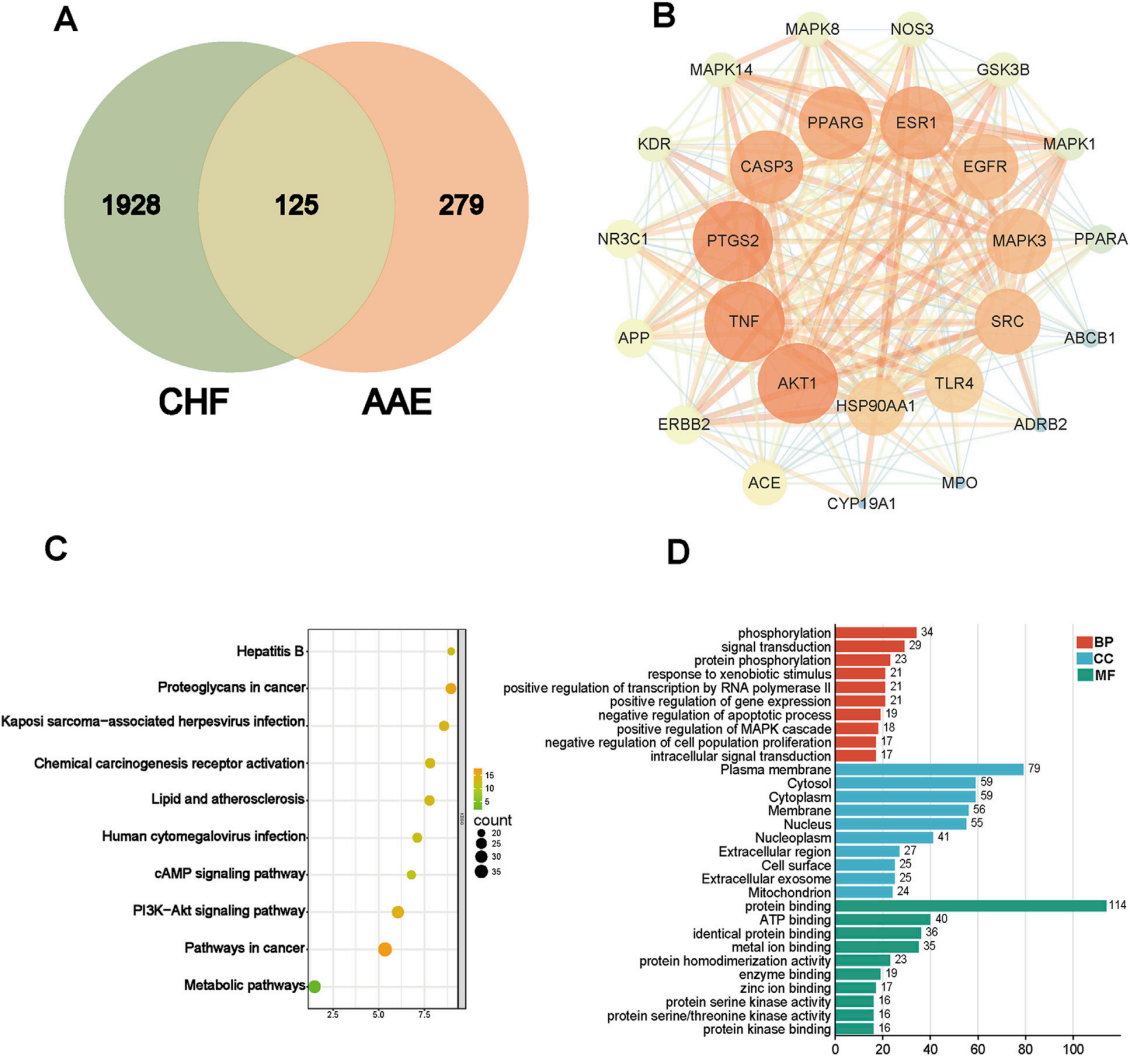
Target prediction via network pharmacology. **(A)** Venn image of drug and disease. **(B)** PPI network of target genes. **(C)** KEGG analysis. **(D)** GO annotations.

We used network analysis to predict potential targets. A total of 404 drug targets were collected by target prediction, and 2053 target genes were filtered via different databases. A total of 125 common targets were obtained through intersection analysis (Figure 2A). And PPI network suggested the participation of AKT in CHF (Figure 2B). KEGG analysis revealed that the PI3K/AKT signaling pathway was involved in CHF (Figure 2C). GO annotation suggested that intersecting genes were mainly enriched in phosphorylation, signal transduction, protein phosphorylation, response to xenobiotic stimulus, positive regulation of MAPK cascade, negative regulation of cell population proliferation, and intracellular signal transduction (Figure 2D).

### 3.3 AEA improved cardiac function in CHF model mice

**FIGURE 3:**
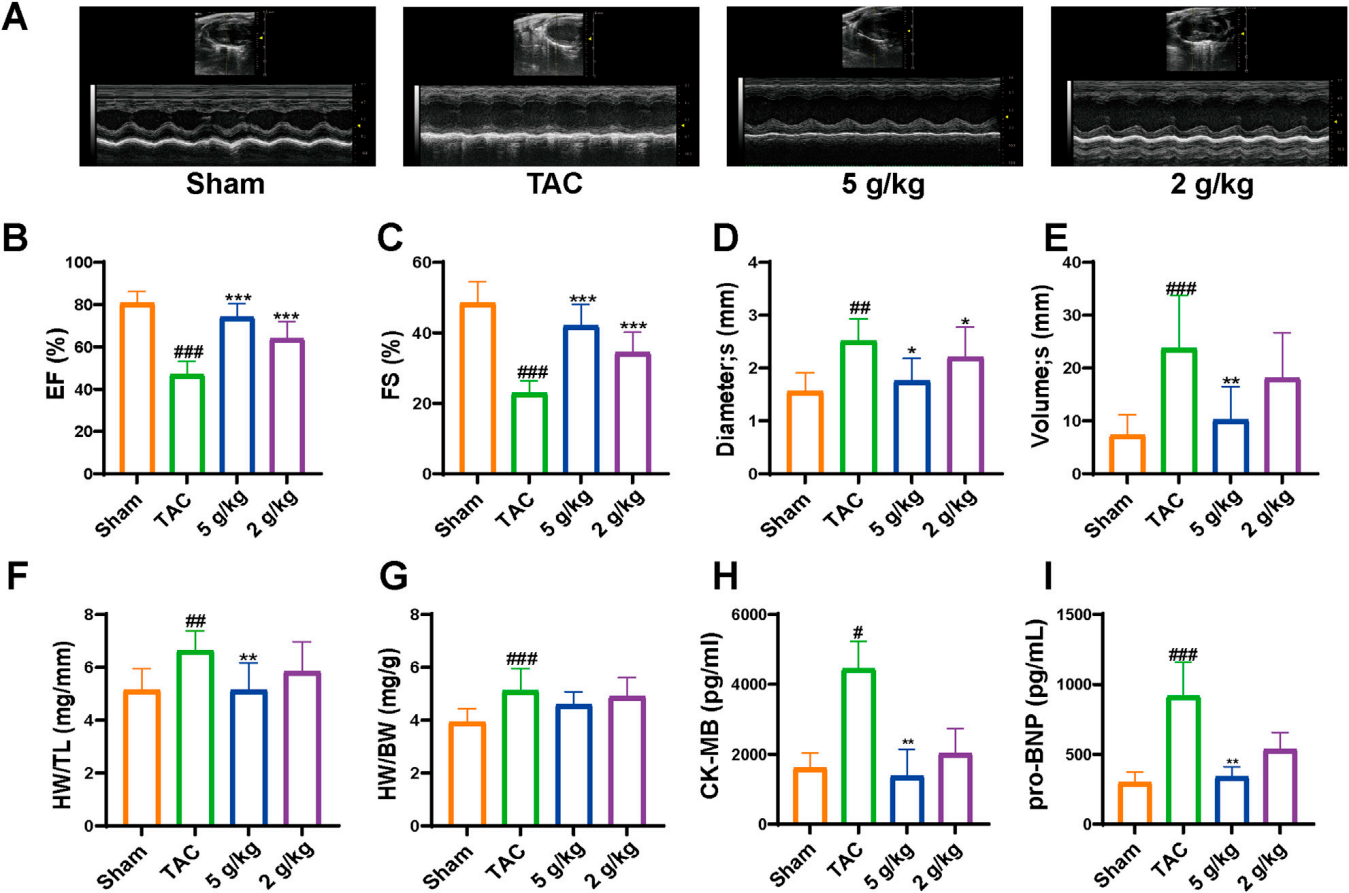
AEA improved cardiac functions. **(A)** The representative images of echocardiography. **(B–E)** The values of diameter (s), volume (s), EF and FS. **(F)** The ratio of heart weight and tibia length. **(G)** The ratio of heart weight to body weight. **(H, I)** ELISA analysis of CK-MB and NT-proBNP. (n = 10). ^#^ *p* < 0.05, ^##^ *p* < 0.01, ^###^ *p* < 0.001 vs. Sham group; ^*^ *p* < 0.05, ^**^ *p* < 0.01, ^***^ *p* < 0.001vs. TAC group.

At 4 weeks, we assessed cardiac function in different groups. The results indicated abnormal changes in cardiac function and morphology. Ejection fraction (EF) is an important index to evaluate systolic function. Compared with that in the Sham group, EF was significantly decreased in the TAC group. AEA administration significantly increased EF in a dose-dependent manner. (Figures 3A, B). Fractional shortening (FS) is usually used to evaluate the cardiac contractility. In this study, we measured FS value and the results indicated a significant change in cardiac systolic function. FS value was significantly decreased in TAC mice, relative to the Sham group. However, the AEA administration partially reversed such results. (Figure 3C). In addition, echocardiography results revealed an increase in diameter and volume at the end of systole, suggesting enlargement of the left chamber. (Figures 3D, E). These results indicated that TAC surgery could affect cardiac function and cause CHF. Long-term use of AEA could optimize cardiac performance to a certain extent, and the therapeutic effect was dose-dependent. To better reflect cardiac hypertrophy, we calculated the ratios of heart weight/tibia length (HW/HL) and heart weight/body weight (HW/BW). There were significant differences between the Sham and TAC groups. Oral administration of AEA suppressed heart mass and reduced the HW/HL ratio (Figures 3F, G). CK/MB and NT-proBnp are important factors for measuring myocardial injury. CK/MB reflects cardiac damage, whereas NT-proBnp is associated with myocardial hypertrophy. Our results revealed that these factors were increased in the TAC group, and AEA treatment partially reversed such results. (Figures 3H, I).

### 3.4 AEA ameliorated cardiac remodeling

**FIGURE 4:**
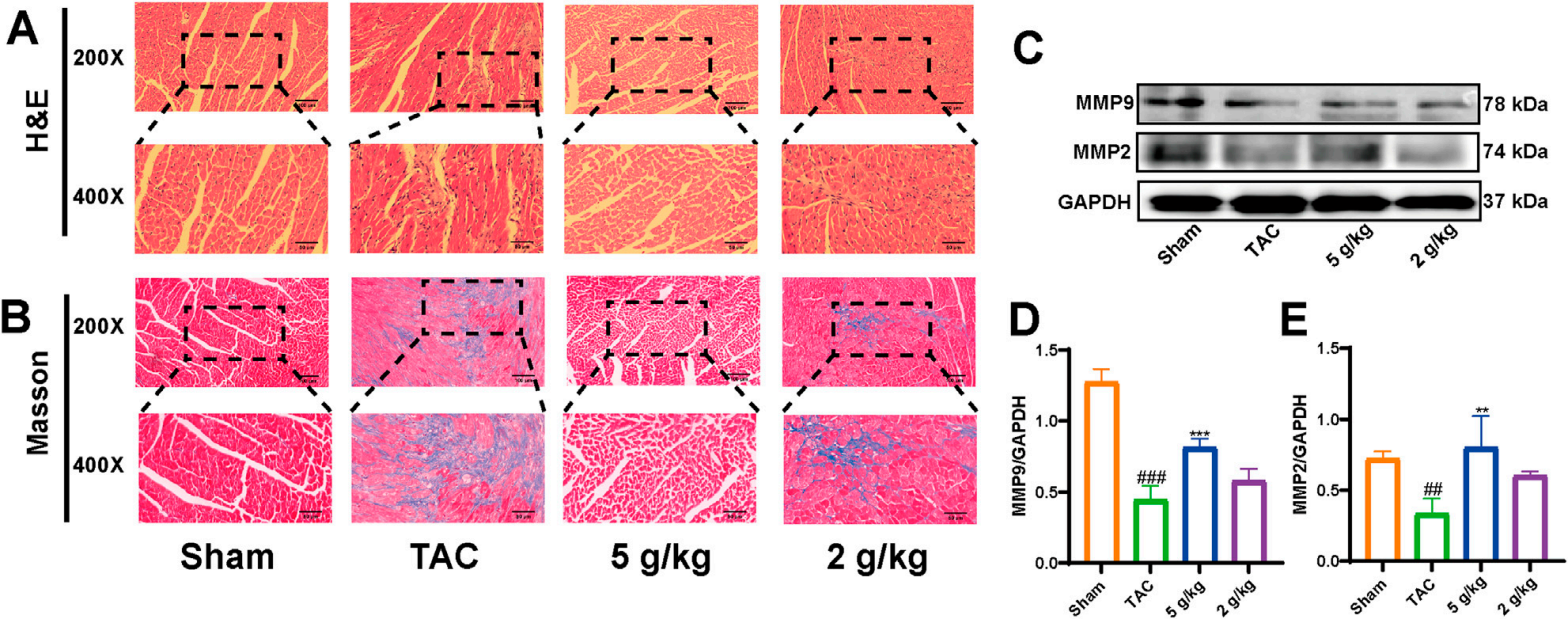
AEA inhibited cardiac remodeling. **(A)** The representative images of H&E staining. **(B)** The representative images of Masson’s staining. (n = 5) **(C, D, E)** The expression of MMP2 and MM9. (n = 3).

H&E staining revealed that the myocardial fibers were arranged neatly, without obvious interstitial fibrosis in the Sham group. However, myocardial fibers were significantly disordered with obvious collagen fiber deposition in the TAC group (Figures 4A, B). Matrix metalloproteinase (MMP) participates in extracellular matrix deposition (Buckley et al., 2023). In addition, we evaluated the expression of MMP2 and MMP9, which were decreased in the TAC group, compared with the Sham and AEA groups (Figures 4C–E).

### 3.5 Effects of AEA intake on serum metabolism

**FIGURE 5:**
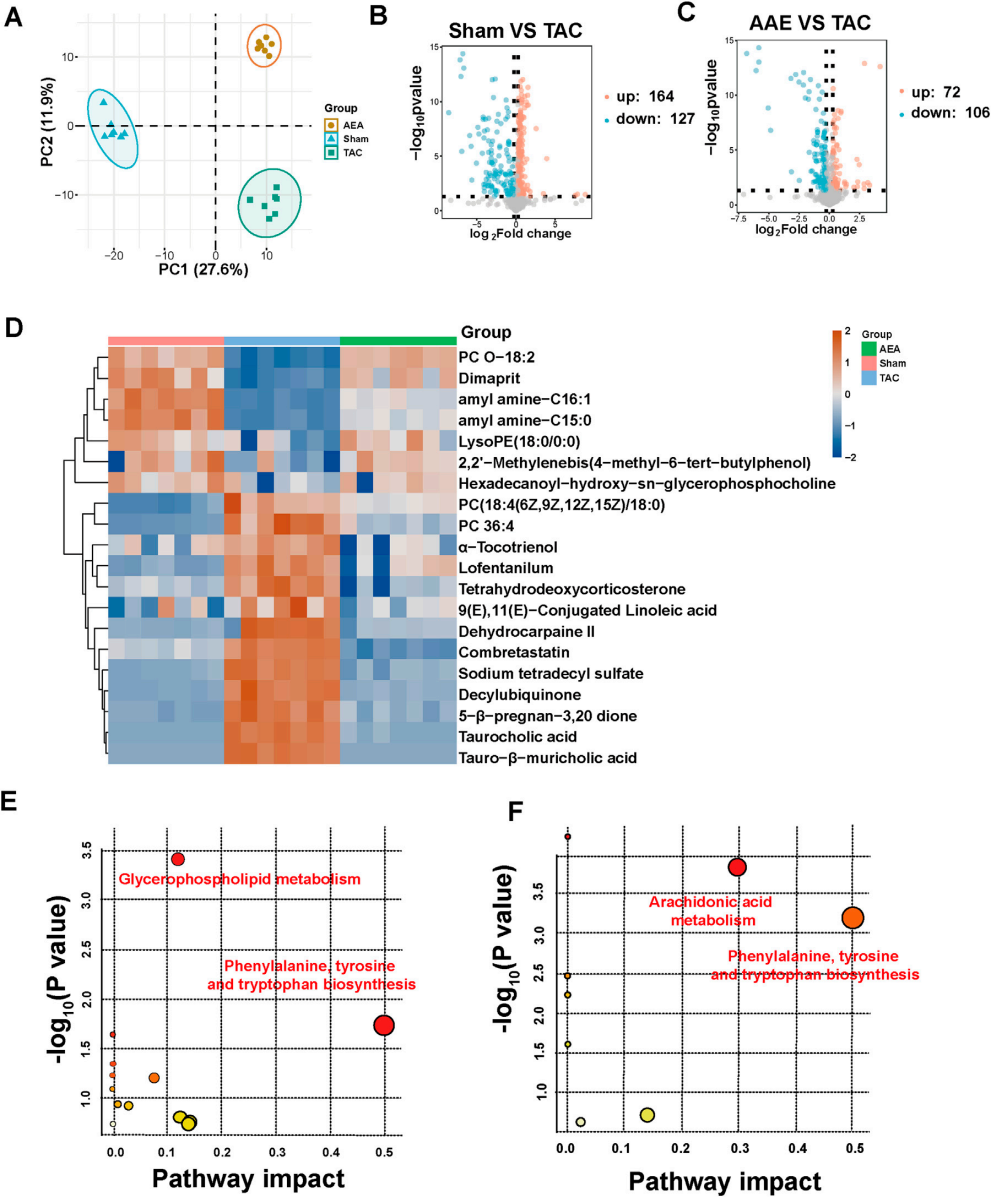
Metabolomics analysis of serum sample. **(A)** PCA analysis of Sham, TAC and AEA groups. **(B)** Volcano plots of Sham and TAC groups. **(C)** Volcano plots of AEA and TAC groups. **(D)** Heat map of partial metabolites. **(E)** The metabolic pathway between Sham and TAC groups. **(F)** The metabolic pathway between AEA and TAC groups. (n = 7).

**FIGURE 6:**
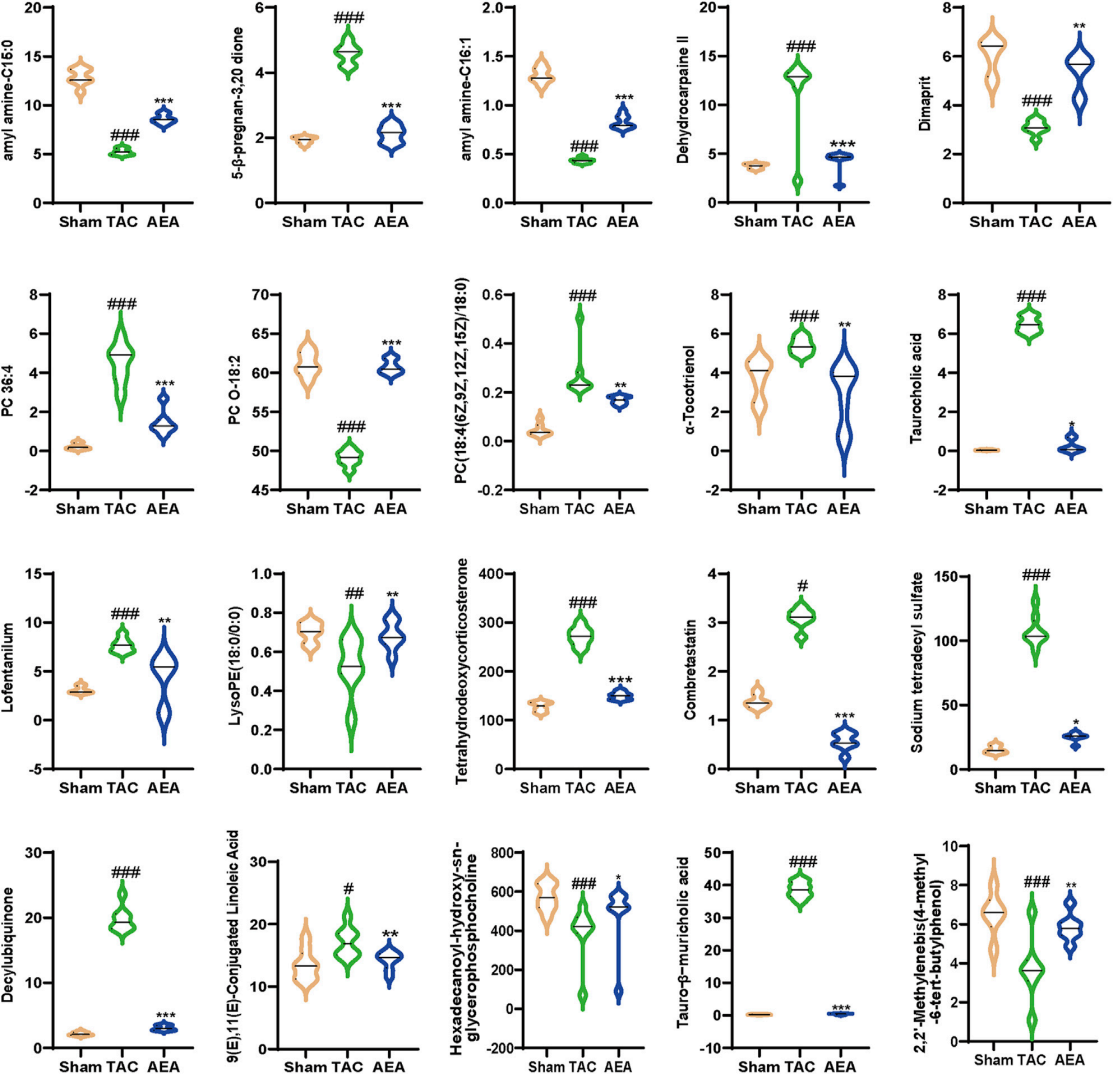
Statistics analysis of differential metabolites. (n = 7).

We used nontargeted metabolomics to investigate the differences in serum metabolites among the Sham, TAC, and AEA groups. PCA analysis revealed that these three groups could be completely separated, indicating differences in metabolite level. The separation between the TAC and Sham groups indicated the success in surgery and pathological changes in mice. Meanwhile, samples from the AEA group were completely separated from those of the TAC group, indicating metabolic differences upon AEA administration (Figure 5A). We subsequently created a volcano map to show specific metabolites. A total of 164 metabolites were upregulated, and 127 metabolites were downregulated in the Sham group, compared with the TAC group (Figure 5B). Moreover, compared with the TAC group, 72 metabolites were upregulated, and 106 substances were downregulated (Figure 5C). A total of 20 metabolites for which *P < 0.05* were selected (Figure 5D). Through metabolic pathways, we found that phenylalanine, tyrosine, and tryptophan biosynthesis was comprised both in Sham VS TAC and AEA VS TAC. While glycerophospholipid metabolism was different between the Sham and TAC groups, arachidonic acid metabolism was different in the AEA and TAC groups (Figures 5E, F). Among them, there were significant differences in several metabolites such as tetrahydroeoxycorticosterone, decylubiquinone, and tauro-*β*-muricholic acid (Figure 6). In particular, tetrahydrooxycorticosterone is a type of mineralocorticoid, and its expression reflects blood pressure. Decylubiquinone is an analog of ubiquinone that inhibits ROS generation. These results indicated metabolic differences among the Sham, TAC, and AEA groups.

### 3.6 AEA regulated the PI3K/AKT/Bnip3 axis and improved cardiac function

**FIGURE 7:**
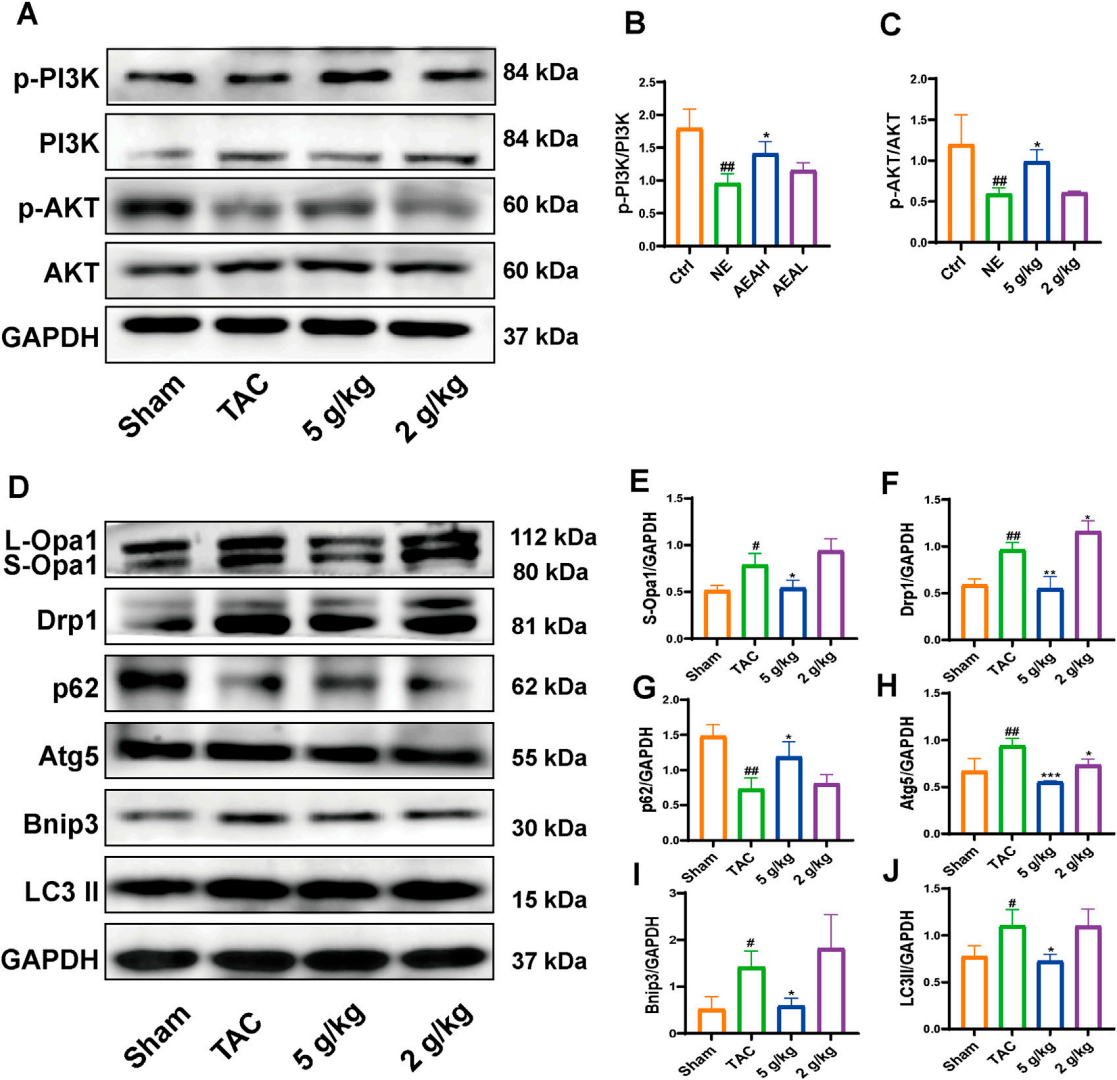
AEA improved CHF via PI3K/AKT/Bnip3 axis. **(A)** Representative images of PI3K/AKT axis. **(B, C)** The phosphorylation level of PI3K and AKT. **(D)** Representative images of Opa1, Drp1, Bnip3, p62, Atg5 and LC3II. **(E–J)** The expression level of Opa1, Drp1, Bnip3, p62, Atg5 and LC3II. (n = 3).

**FIGURE 8:**
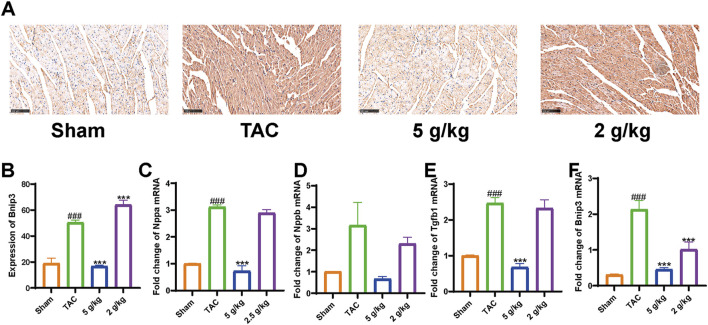
AEA suppressed elevation of Bnip3. **(A)** Immunohistochemistry of Bnip3. **(B–F)** Expression of Nppa, Nppb, Tgfb1, and Bnip3 mRNA. (n = 3).

We used Western blotting to measure protein levels in the PI3K/AKT/Bnip3 pathway. By measuring p-PI3K, PI3K, p-AKT, and AKT levels in cardiac tissue, we found that the phosphorylation of PI3K and AKT was decreased, relative to the Sham group. However, AEA administration increased the phosphorylation of these proteins to some extent. (Figures 7A–C). We investigated fission by measuring the expression of S-Opa1 and Drp1. The ratios of S-Opa1/GAPDH and Drp1/GAPDH were increased in the TAC group. AEA administration inhibited the increase of S-Opa1 and Drp1 and suppressed mitochondrial fission. (Figures 7D–F). Bnip3 is an OMM receptor, and its abnormal expression is associated with apoptosis and mitophagy. In this experiment, we mainly focused on the effects of mitophagy. To investigate Bnip3-mediated mitophagy, we evaluated proteins associated with mitophagy, such as Atg5, p62, and LC3 II (Figures 7G–J). The results indicated that sustainable constriction triggered mitophagy, manifested as the increase of Atg5 and LC3 II, and a decrease of p62. Oral administration of AEA inhibited mitochondrial damage and suppressed Bnip3-mediated mitophagy. We used immunohistochemistry to determine the content of Bnip3 in the sections and found that the TAC group was significantly increased (Figures 8A, B). Additionally, the mRNA expression of Nppa, Nppb, Tgfb1, and Bnip3 was elevated in the TAC group (Figures 8C–F).

### 3.7 AEA ameliorated mitochondrial damage

**FIGURE 9:**
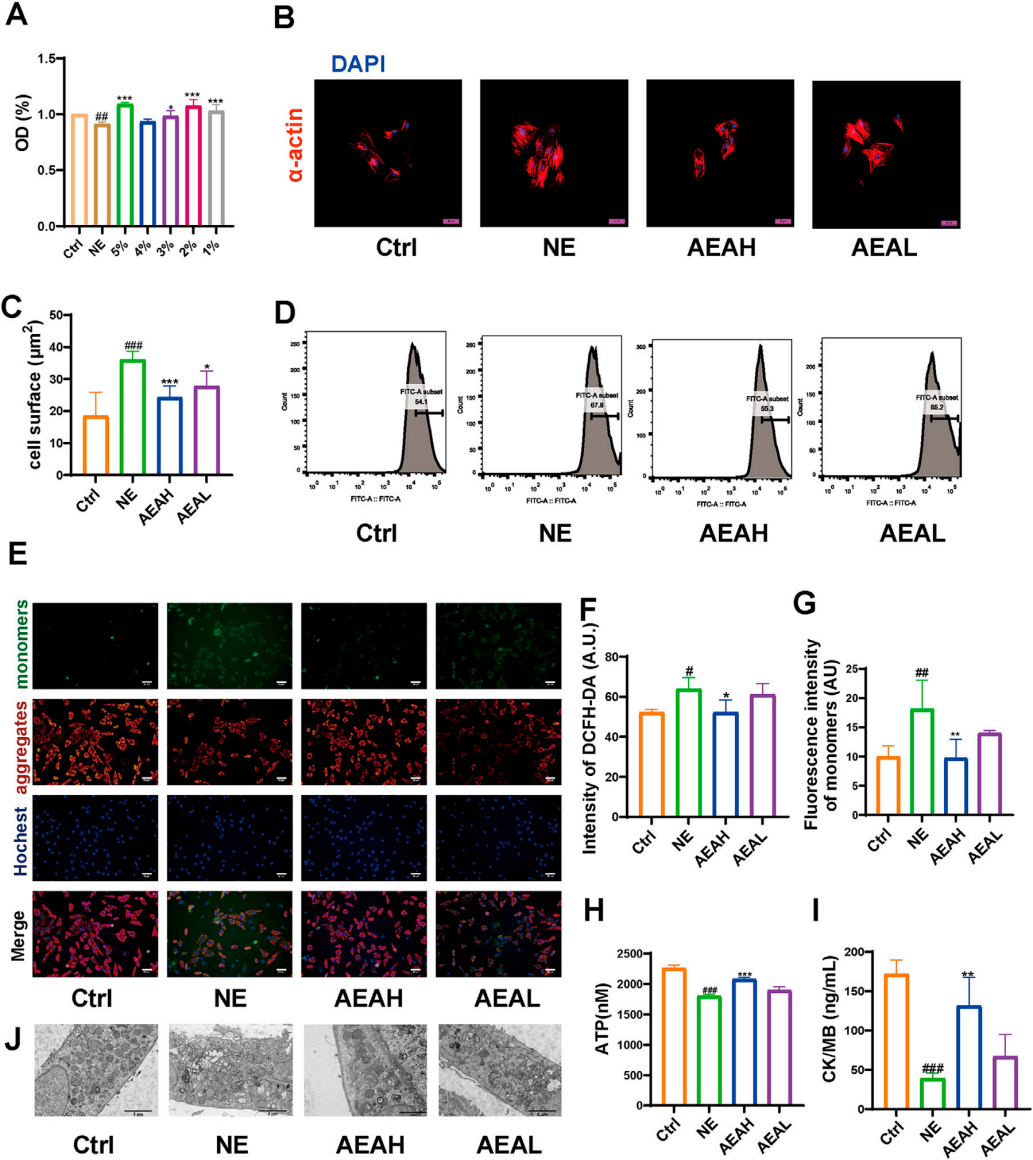
AEA hampered mitochondrial injuries. **(A)** Cell viability of H9c2 cells. **(B)** Fluorescence staining of α-actin on cell surface. **(C)** Statistics analysis of cell surface area. **(D)** Flow cytometry analysis of ROS intensity. **(E)** JC-1 staining of different groups. **(F)** Statistics analysis of DCFH-DA intensity. **(G)** Statistics analysis of monomers intensity. **(H)** ATP content. **(I)** CK/MB content. **(J)** Transmission electron microscope analysis of H9c2 cells. (n = 3).

Mitochondrial damage often triggers mitophagy. To mimic abnormal changes in CHF, we stimulated H9c2 cells with 50 μM NE and measured cell surface area, ROS level, mitochondrial membrane potential, ATP concentration, and CK/MB content. We conducted MTT experiments to obtain appropriate concentration and selected 1% as the high dose and 0.5% as the low dose. α-actin sustaining showed that the cell surface area was elevated in the NE group, compared to the Ctrl group, whereas the area was decreased after AEA administration. (Figures 9A–C). Excessive ROS decrease the cellular antioxidant capacity and facilitate mitochondrial damage. We conducted flow cytometry to assess the changes in ROS level. It was displayed that ROS content was elevated in the NE group, and AEA treatment reduced ROS generation. (Figures 9D, F). Mitochondrial membrane potential is vital for maintaining ROS generation. In our study, we sustained the mitochondrial membrane potential with JC-1 dyes. The fluorescence intensity of the monomers decreased, manifesting mitochondrial damage, while AEA administration partially reversed such result (Figures 9E, G). Additionally, we used an enhanced kit to measure ATP content in H9c2 cells. The results indicated that NE could reduce ATP generation and AEA could increase ATP levels. (Figure 9H). CK/MB is associated with ROS generation (Keceli et al., 2022). In addition, we measured CK/MB levels in mitochondria. This factor was elevated in the NE group, compared with the Ctrl and AEA groups (Figure 9I). Additionally, transmission electron microscope results indicated a destruction in the mitochondria structure upon NE treatment (Figure 9J).

### 3.8 AEA ameliorated mitochondrial damage via PI3K/AKT/Bnip3 axis

**FIGURE 10:**
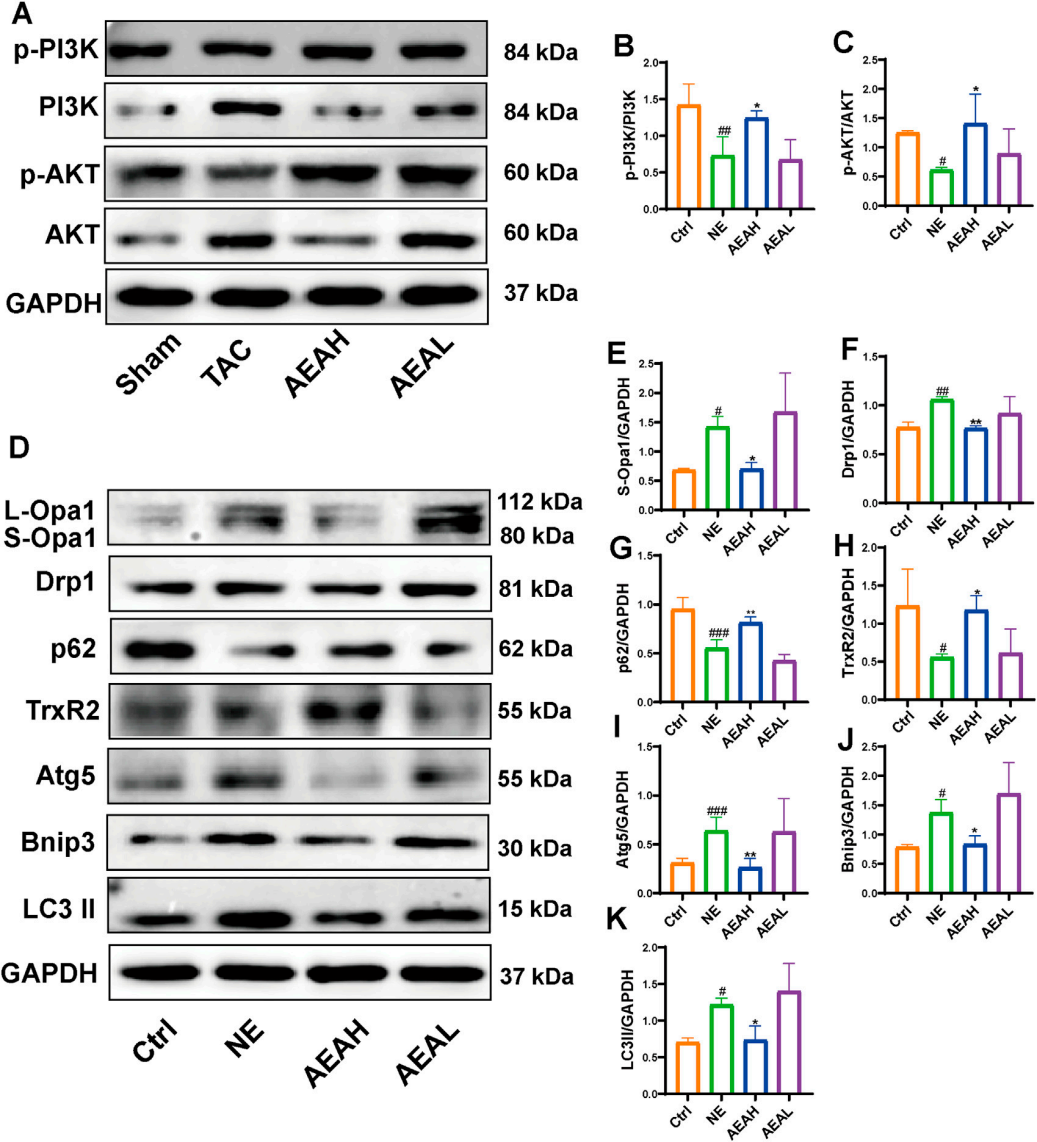
AEA improved NE-induced injuries via PI3K/AKT/Bnip3 axis. **(A)** Representative images of PI3K/AKT axis in H9c2 cells. **(B, C)** The phosphorylation level of PI3K and AKT. **(D)** Representative images of Opa1, Drp1, TrxR2, Bnip3, p62, Atg5 and LC3II in cells. **(E–K)** The expression level of Opa1, Drp1, TrxR2, Bnip3, p62, Atg5 and LC3II in H9c2 cells. (n = 3).

**FIGURE 11:**
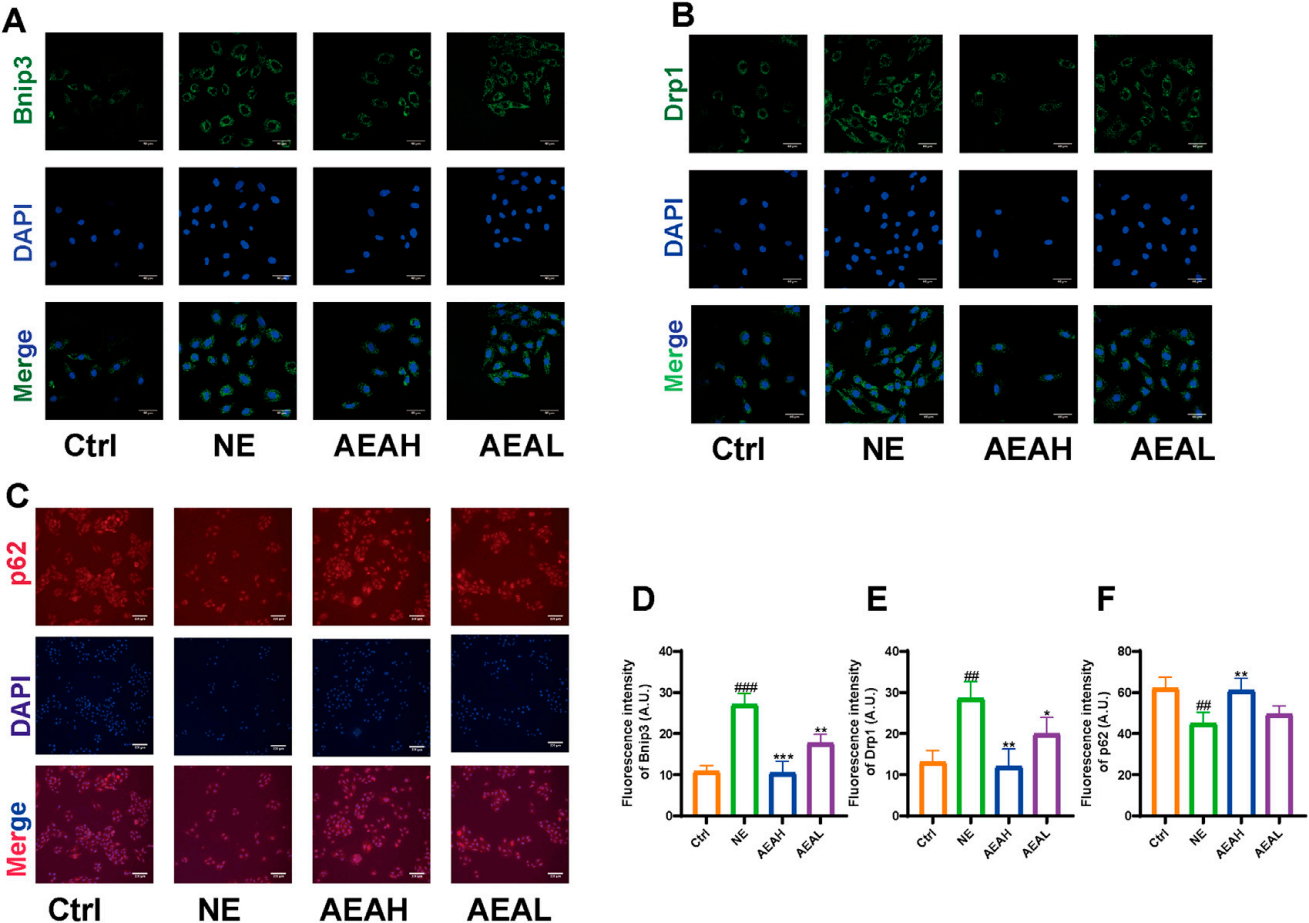
Immunofluorescence of Bnip3, Drp1, and p62 in H9c2 cells. **(A)** Immunofluorescence of Bnip3. **(B)** Immunofluorescence of Drp1. **(C)** Immunofluorescence of p62. **(D)** Quantitative analysis of Bnip3. **(E)** Quantitative analysis of Drp1. **(F)** Quantitative analysis of p62. (n = 3).

**FIGURE 12:**
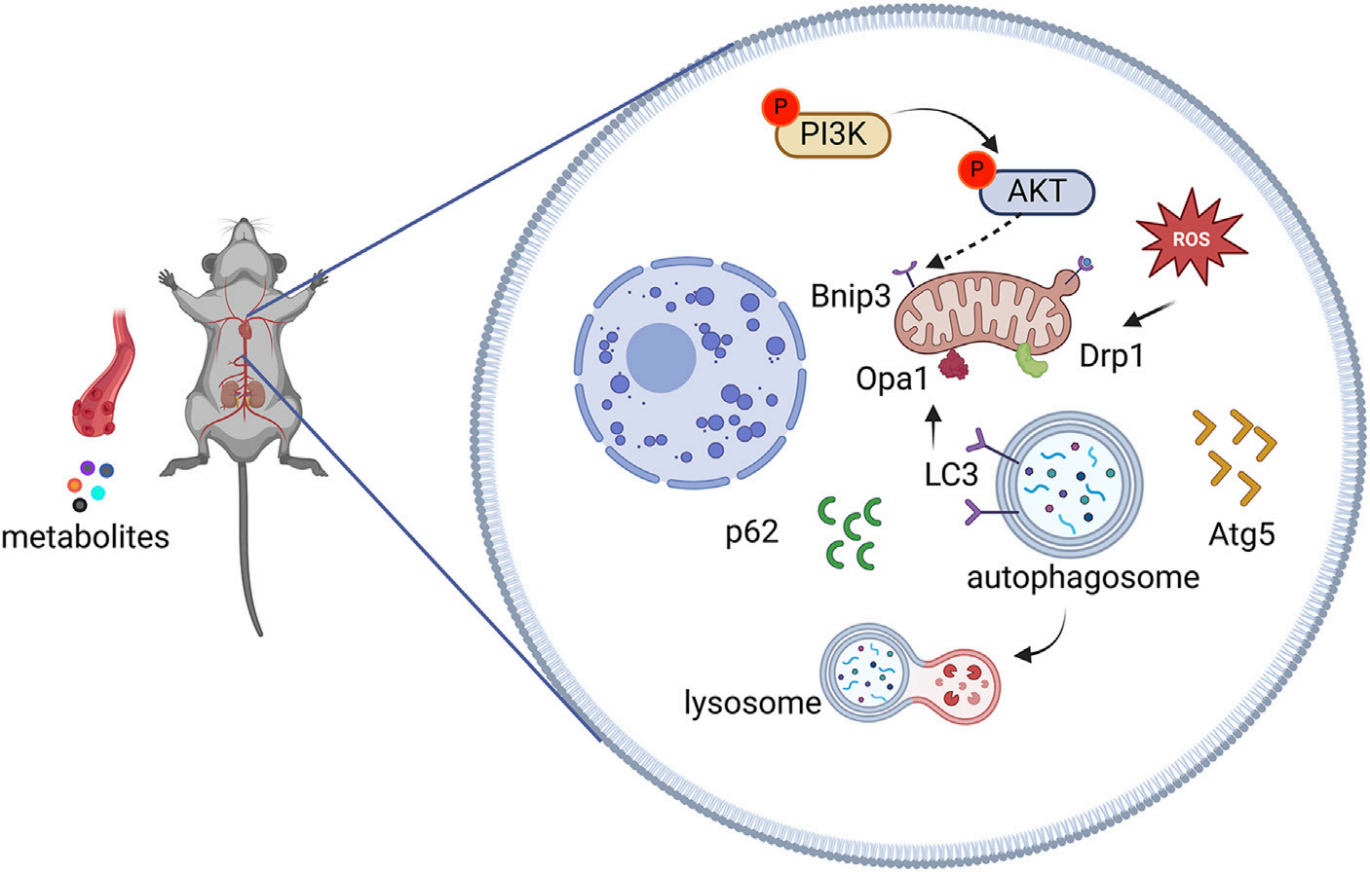
Effects of AEA on chronic heart failure via PI3K/AKT/Bnip3 pathway.

To determine whether AEA could mitigate mitophagy caused by NE, we conducted WB to evaluate protein levels in H9c2 cells. Our results indicated that phosphorylation levels of PI3K and AKT were reduced in the NE group, and AEA administration partially reversed such results. Opa1 and Drp1 are associated with mitochondrial fission. We evaluated the levels of Opa1 and Drp1 in H9c2 cells, and the results found that both proteins were elevated in the NE group. (Figures 10A–F). Txr2R is a mitochondrial thioredoxin reductase related to ROS release (Stanley et al., 2011). The expression was downregulated in NE, compared with Ctrl and AEA groups (Figure 10G). To investigate the changes in mitophagy, we detected the content of Bnip3, Atg5, LC3II, and p62. The results showed that compared with the Sham group, mitophagy level in the NE group was elevated, as manifested by increases of Bnip3, Atg5, and LC3II, and a decrease of p62 (Figures 10H–J). Moreover, the immunofluorescence results in H9c2 cells indicated an elevation of Bnip3 and Drp1 in the NE group, as well as a decrease of p62 (Figure 11). The detailed mechanism is displayed in Figure 12.

## 4 Discussion

CHF is a clinical syndrome that threatens human health (Li et al., 2025). The incidence has increased exponentially with the development of an aging society (Alfaro et al., 2025). Aconiti Lateralis Radix Praeparata was first recorded in the *Shennong Classic of Materia Medica,* and it was frequently used in ancient China to treat heart failure (Zhang et al., 2022). However, the mechanism was ambiguous, necessitating further exploration. In this study, AEA ameliorated CHF by affecting PI3K/AKT/Bnip3 axis. Network analysis was used to predict targets, and some genes were enriched in the PI3K/AKT pathway. TAC surgery was conducted to induce CHF, and different concentrations of AEA were orally administered for 21 consecutive days. Four weeks was chosen as the experimental duration. At this time, cardiac function was abnormal, accompanied by mitochondrial damage. The results indicated that AEA could mitigate Bnip3-mediated mitophagy by altering the phosphorylation of PI3K and AKT.

Mitochondria play important roles in oxidative phosphorylation, electron transport, calcium ion balance, and signal transduction (Huang et al., 2024). Upon adverse stimulation, mitochondria suffer from ROS accumulation, membrane potential decrease, mitochondrial DNA damage, and unfolded protein responses. ROS are generated by normal or damaged mitochondria, and excessive ROS induce oxidative stress (Di Rienzo et al., 2024). ROS plays an intricate role in mitochondria, along with the complex interactions between different parameters, remain confusing points that warrant further exploration (Lin et al., 2025). Cardiac damage caused by ROS has become a hot topic, and emerging studies have explored antioxidant drugs based on the characteristics of CHF (Liu, Huang, et al., 2025). We observed an elevation of ROS upon NE stimulation, which was partially reversed upon AEA treatment. It is well known that mitochondrial membrane potential is associated with ROS generation (Zhao et al., 2025). Loss of mitochondrial membrane potential causes autophosphorylation of proteins, promotes autophagosome recruitment, as well as facilitates mitophagy (Lv et al., 2024). Moreover, sustained stimulation leads to ROS elevation, thereby exacerbating mitochondrial damage and cardiac injury. In our study, the fluorescence intensity of monomers in the NE group was elevated, suggesting a loss of membrane potential. ATP is the source of energy. The influence of ATP is contingent upon the concentration and conversion (Yang et al., 2025). It has been observed that ATP diminishment might precede mitophagy, and excessive mitophagy would precipitate such loss (Lu et al., 2025). Thus, we detected ATP concentration in H9c2 cells, and the results indicated a disruption of ATP generation. Herein, we found that AEA could ameliorate mitochondrial injuries and facilitate energy metabolism in myocardial tissue with chronic stimulation. CK/MB reflects myocardial injury (Zheng et al., 2025). Subsequently, we delved into the difference between the serum and mitochondria. we detected CK/MB in mitochondria via an isolation kit. Briefly, mitochondria were extracted from H9c2 cells. Thus, the CK/MB level in mitochondria rather than the whole cell was measured. Besides changes in mice serum, CK/MB content decreased in mitochondria, which means the destruction of the outer membrane. Therefore, we proposed that NE could affect normal mitochondrial functions. However, these effects were partially counteracted by the AEA administration.

Mitochondrial dynamics involves fusion, fission, biogenesis, and mitophagy (Zhang Z. Y. et al., 2024). Mitochondrial fission refers to the process in which mitochondria divide into two or more smaller organelles, thus ensuring sufficient numbers to maintain normal functions upon adverse stimulation (Chen and Chan, 2009). This process is mediated by a GTPase protein Drp1. During mitochondrial fission, Drp1 forms oligomers on the outer membrane, leading to mitochondrial narrowing and separation (Sami Alkafaas et al., 2024). Mitochondria are conducive to phagocytosis after fragmentation (Martinvalet, 2018). Research has shown that mitochondria are prone to fragmentation in individuals with adverse conditions such as diabetic cardiomyopathy, septic cardiomyopathy, ischemia/reperfusion injury, and myocardial infarction (Rahmani et al., 2024). Based on previous experiments, we speculated that TAC stimulated mitochondrial fission (Liu, Wang, et al., 2025). We measured Drp1 expression in different groups, and the results revealed an elevation in the TAC group. Mitochondrial fusion is the opposite process of mitochondrial fission and refers to the fusion of different mitochondria (Murata et al., 2024). What’s more, mitochondrial fusion plays a protective role by inhibiting mitochondrial fission and restoring the mitochondrial membrane potential upon streptozotocin stimulation (Wang et al., 2025b). Different from fission, mitochondrial fusion is mediated by Opa1, Mfn1, and Mfn2, respectively (Liu et al., 2025a; Liu et al., 2025b; Meng et al., 2024). Numerous studies highlighted the crucial role of Opa1 in mitochondrial dynamics (Liu, Yin, et al., 2025). Opa1 is a dynamin-like GTPase located in the inner membrane that exerts a critical role in cristae (Liang et al., 2024). Upon a decrease in the mitochondrial membrane, L-Opa1 was proteolytically cleaved into S-type by metalloprotease (Garcia et al., 2021). This process caused a collapse of the mitochondrial network and promoted cell apoptosis. Moreover, S-type Opa1 is tightly related to mitochondrial fission (Zhang et al., 2020). We focused on the relationship between mitochondrial fission and mitophagy. By measuring the S-Opa1 level, we observed a division in mitochondria, as the higher S-Opa/GAPDH ratio in the TAC and NE groups indicated the enzymatic hydrolysis of L-Opa1. The above results indicated an enhancement of mitochondrial fission.

Mitophagy is common in CHF. Mitophagy often occurs in response to adverse stimuli such as mitochondrial depolarization, nutrient deficiency, ROS accumulation, and cellular aging (Xu et al., 2024). The process can be divided into two categories, and Bnip3 is included in the receptor-mediated mitophagy (Tang et al., 2024). Bnip3 is located on the outer mitochondrial membrane and has a BH3 domain (Su et al., 2023). Bnip3 often exists as a dimer, and excessive monomers are degraded by proteasomes or lysosomes (Delgado and Shoemaker, 2024). Notably, Bnip3 recruits LC3 protein through the LIR region and binds to autophagosomes for further elimination (Delgado et al., 2024). Moreover, Bnip3 regulates the connection between mitochondria and proapoptotic proteins and exhibits a crucial role in cell apoptosis (Deng et al., 2024). In addition, Bnip3 also serves as a sensor to convert oxidative stress signals into apoptotic signals and transmit them to mitochondria (Xie et al., 2024). Regarding the relationship between Bnip3 and mitophagy, Bnip3 activates mitophagy without the involvement of E3 enzymes (Degli Esposti, 2024). It often binds to autophagosomes through the LIR region (Rahman et al., 2024). Mature autophagosomes then fuse with lysosomes, leading to the degradation of mitochondria (Feng et al., 2024). In this study, we detected Bnip3 levels in different groups, and the results indicated an elevation in the TAC and NE groups. To reflect mitophagy levels in CHF, we assessed the expression of Atg5, p62, and LC3II via Western blotting. It was shown that Atg5 and LC3II were increased in TAC and NE groups, whereas AEA treatment suppressed the activation of mitophagy and ameliorated CHF.

PI3K is a lipid kinase that comprises three subunits: p55, p58, and p110 (Gutiérrez-Vázquez et al., 2017). When PI3K is activated, the p110 subunit catalyzes the transformation of phosphatidylinositol-4,5-disodium diphosphate (PIP2) into phosphatidylinositol-3,4,5-triphosphate (PIP3), and recruits AKT (Zhang Z. Y. et al., 2024). AKT protein is a serine/threonine kinase with three isoforms: AKT1, AKT2, and AKT3 (Adon et al., 2025). Among them, AKT1 has been mostly studied in different fields. Typically, PIP3 activates AKT protein by phosphorylating amino acid residues 473 and 308, establishing a link between PI3K and AKT (Zhao et al., 2024). Our network analysis indicated that AKT1 was among the intersection genes, and participated in the CHF. Common downstream genes of AKT such as FOXO, mTOR, HIF-α, and MAPK proteins, are involved in heart diseases, and are associated with mitophagy (Zheng et al., 2024). Furthermore, PI3K affects the occurrence of autophagy via a complex of PI3K, Beclin1, VSP15, VSP34, and ATG14L (Liang et al., 2020). Our KEGG analysis indicated a participation of PI3K/AKT in CHF. Therefore, we speculated that the PI3K/AKT/Bnip3 axis was involved in AEA mechanisms. Our WB results indicated a decrease in AKT phosphorylation. Moreover, AEA administration increased AKT phosphorylation and inhibited Bnip3-mediated mitophagy.

Tetrahydroxycorticosterone is a type of mineralocorticoid produced by the adrenal gland and acts as a precursor to aldosterone (Gorsline et al., 1985). Deoxycorticosterone is mainly metabolized in the liver to produce aldosterone, as well as stimulated sodium reabsorption in renal tubules (Uijl et al., 2021). Excessive aldosterone promotes functional disorders such as inflammation and fibrosis in the kidneys, heart, and cardiovascular system (Kang et al., 2024). Aldosterone level was elevated in numerous diseases (Bakris, 2024). Compelling evidence has shown that aldosterone is associated with diastolic dysfunction (Ueda et al., 2022). These results were consistent with our findings. The tetrahydrofuran level was significantly increased in the mice serum from TAC group, indicating the occurrence of myocardial remodeling and cardiac disorders.

Decylubiquinone is an FDA-approved drug that inhibits cytochrome C release by altering mitochondrial permeability (Cao et al., 2020). Decylubiquinone is an analog of coenzyme Q10, that exists in the mitochondria. It is an important component of the oxidative phosphorylation system (Iglesias-Romero et al., 2024). Coenzyme Q10 is primarily present in the liver, heart, and kidneys. Elevated levels in plasma indicate mitochondrial damage, leading to the leakage of coenzyme Q10 (Hooshangi Shayesteh et al., 2024). Our omics results showed that compared with that in the Sham group, decylubiquinone content in mice serum was elevated. Moreover, AEA administration partially lowered the concentration of decylubiquinone, and the peak area was significantly decreased.

In summary, AEA effectively protected against CHF. Network analysis was used to predict targets, and metabolomics provided valuable insights into changes in metabolites. In particular, the attenuation of mitochondrial damage and consequent suppression of mitophagy represent key mechanisms contributing to the protective role of AEA against CHF. However, some limitations exist in this study. The cell line used in the present study was immortalized, it is necessary to extract primary cells from heart tissue and perform associated experiments on different cell types. Additionally, the mechanisms we identified in the present study were predicted based on network analysis. We tried to relate these targets with metabolomics, but the PI3K/AKT axis was not included in the metabolic pathway. Thus, we focused on specific metabolites rather than the whole pathway. Furthermore, a PI3K inhibitor should be used to treat H9c2 cells, as this may help to confirm the effect of AEA on PI3K.

## 5 Conclusion

In this study, we found that Aconiti Lateralis Radix Praeparata could ameliorate CHF via the PI3K/AKT/Bnip3 axis. By measuring cardiac parameters, we found that long-term use of AEA improved heart function, prevented excessive mitochondrial engulfment, and improved mitochondrial quality. However, some shortcomings persisted in experiments, such as a lack of multiomics and the deficiency of inhibitors.

## Data Availability

The raw data supporting the conclusions of this article will be made available by the authors, without undue reservation.

## Abbreviations

CHF, chronic heart failure; TAC, transverse aortic constriction; NE, norepinephrine; ROS, reactive oxygen species; AEA, aqueous extract of Aconiti Lateralis Radix Praeparata; EF, ejection fraction; FS, fractional shortening; TCM, traditional Chinese medicine; Bnip3, Bcl-2/adenovirus E1B 19 kDa protein-interacting protein 3.
